# Values and Preferences of Preterm Infants’ Health Outcomes from the Perspective of Parents, Healthcare Providers, and Children or Adults Born Preterm: A Systematic Review

**DOI:** 10.1159/neo/adsag012

**Published:** 2026-09-08

**Authors:** Martin Ringsten, Kenneth Färnqvist, Vera Wachtmeister, Katarzyna Wróblewska-Seniuk, Matteo Bruschettini, María Ximena Rojas-Reyes, Isolde Sommer

**Affiliations:** a Cochrane Sweden, Department of Research, Development, Education and Innovation, Skåne University Hospital, Lund University, Lund, Sweden; b Karolinska Institutet, Department of Molecular Medicine and Surgery, Stockholm, Sweden; c Karolinska Institutet, Department of Clinical Neuroscience, Stockholm, Sweden; d II Department of Neonatology, Poznan University of Medical Sciences, Poznan, Poland; e Paediatrics, Department of Clinical Sciences, Lund University, Lund, Sweden; f Iberoamerican Cochrane Center, Barcelona, Spain; g Cochrane Austria, Department for Evidence-based Medicine and Evaluation, University for Continuing Education Krems, Krems, Austria

**Keywords:** Outcomes, Values, Preferences, Preterm, Systematic review

## Abstract

**Introduction:**

Identifying values and preferences regarding health outcomes for preterm infants is a key aspect of informing clinical decision-making and guideline development.

**Methods:**

We searched for quantitative and qualitative studies conducted among parents, healthcare professionals, and individuals born preterm, assessing the values regarding mortality, clinical outcomes, and long-term disabilities. Screening, data extraction, critical appraisal, and certainty of evidence assessments were performed in duplicate.

**Results:**

We included 24 studies, 16 quantitative and 8 qualitative, including 4,613 participants. Very low certainty evidence suggests that survival is the most valued outcome for parents and individuals born preterm. In contrast healthcare professionals assigned a lower value to survival when it was associated with the risk of disability and poor quality of life. Very severe disability, especially cognitive impairment, was considered less desirable than survival across all groups, but with variation within each group. Very low certainty evidence indicates that among healthcare professionals, intraventricular hemorrhage is the most severe and undesirable condition in the short term, together with suffering and pain. Evidence is very uncertain regarding how values on the same outcomes differ among families, individuals, professionals, and clinical contexts. Some outcome preferences may change over time, both in relative importance and across different stages of a child’s development.

**Conclusion:**

The findings can inform clinical decision-making, guideline development, and health professional education related to values and variability of outcomes. However, the uncertainty and variability between and within interest-holder groups should be considered. Future research should use validated measurements and enroll key interest-holders to improve the evidence.

## Introduction

Approximately 10% of all infants are born preterm (<37 weeks’ gestational age), and about 1.5% are born very (28–32 weeks) or extremely preterm (<28 weeks) [1]. The lower the gestational age, the higher the risk of morbidity and the need for neonatal care [2]. To improve survival and reduce morbidity, preterm infants receive many interventions during the neonatal period, which may have both benefits and harm. Balancing the benefits and harms of interventions in these infants can be challenging for clinicians who must make decisions regarding their treatment and integral management. At this stage, consideration of patients’ values and preferences is an indispensable step in clinical decision-making and in guideline development, as people perceive and value health outcomes differently, leading to individual preferences for certain outcomes over others. Furthermore, transparency about the values placed on certain outcomes can facilitate the understanding and acceptance of decisions to implement or use interventions for preterm infants.

Patients’ values and preferences are defined according to the Grading of Recommendations Assessment, Development and Evaluation (GRADE) approach as the relative importance that people place on health outcomes [3]. Health outcomes for children born preterm can include, but are not limited to, mortality and different types of disabilities and impairments, which can be assessed both in the short and long terms. To address this issue in the neonatal field, identifying and synthesizing the values and preferences of individuals born preterm, as well as those of parents of preterm children, and professionals caring for these patients, is needed. We aimed to assess the values and preferences of health outcomes for preterm infants from the perspectives of parents, caregivers, healthcare providers and children or adults born preterm, to inform guideline development for preterm infants.

## Methods

We conducted a mixed-methods systematic review. We adhered to the Preferred Reporting Items for Systematic Reviews and Meta-Analyses (PRISMA) 2020 statement [4] for reporting the findings of this review. We developed an a priori protocol [5] describing the methodology of this review and uploaded it to Open Science Framework.

### Study Eligibility Criteria

We included qualitative or quantitative research studies that assessed the values and preferences of the health outcomes of preterm infants receiving intensive care, with or without ventilatory support. The study populations included children or adults born preterm, caretakers or parents of preterm infants, or healthcare personnel caring for preterm infants. We excluded all studies on values and preferences related to neonatal resuscitation immediately after the birth. Publications had to be peer-reviewed (abstracts, letters, and preprints were excluded). Detailed criteria are presented in online supplementary Table S1 (for all online suppl. material, see https://doi.org/10.1159/neo/adsag012).

### Literature Search

An experienced information specialist from Lund University searched Embase (Embase.com), Medline (via PubMed), Scopus (scopus.com), Web of Science (Core Collection), and PsycInfo (via Ebsco) from inception to 5 November 2025 (latest update search) using MESH and free-text terms. We supplemented searches by screening reference lists of included studies. The full search strategy is available in online supplementary Table S2.

### Study Selection

Two authors assessed each study based on the title and abstract in duplicate, and then the full text (M.F., K.F., V.W., M.X.R., I.S.). Any discrepancies were resolved through discussion between the two reviewers or consultation with a third reviewer. We used Covidence (Veritas Health Innovation, Melbourne, Australia) for literature screening.

### Risk of Bias Assessment, Data Extraction and Certainty of Evidence Assessment

We used the Risk of Bias in Studies of Values and Utilities (ROBVALU) tool [6] for quantitative studies and the Cochrane Qualitative Methodological Limitations Tool [7] for qualitative studies, with assessments conducted in pairs (K.F., I.S.; I.S., V.W.). Discrepancies were resolved through discussions or consultations with a third team member. Data extraction of the characteristics of the study populations, settings, and methods was performed by one reviewer for quantitative (M.R., K.F., V.W., I.S.) and qualitative (V.W., I.S.) studies, with a second reviewer verifying the data abstraction. Two reviewers (M.R., K.F., V.W., I.S.) independently extracted data on the values and preferences of outcomes using a calibrated predesigned form in Excel® software. Conflicts were resolved through discussions or consultations with a third reviewer. We assessed the certainty of evidence following the GRADE for quantitative evidence (Zhang 2019) and using the Confidence in the Evidence from Reviews of Qualitative Research (GRADE-CERQual) approach [8] for qualitative evidence. Initial judgments were made by one reviewer (I.S.), followed by a discussion and consensus with a second reviewer (M.R.).

### Synthesis of Results

We followed a mixed-methods approach to synthesize and integrate quantitative and qualitative evidence whenever possible. The synthesis approach was guided by the convergent integrated approach proposed by the Joanna Briggs Institute (JBI) [9]. Quantitative data were transformed into contextual descriptors and presented with qualitative data during the analysis stage. Where appropriate, values, and preferences were reported by population subgroups (e.g., parents, healthcare professionals, and people born preterm) and included key information on variability across these subgroups.

The final relative importance of the outcome categories was guided by a guideline panel of neonatologists. Outcomes prioritized were:

1. Survival
2. Quality of life (QoL)
3. Developmental impairment (cognitive impairments, cerebral palsy, and mental delay)
4. Other impairments (hearing, visual, motor disabilities)
5. Clinical outcomes (undesirable outcomes commonly associated to the neonatal care)

The variety of methods used among the included studies did not allow the application of statistical methods for synthetizing results for each outcome, and the findings are presented narratively.

## Results

We screened a total of 3,534 records, reviewed 113 in full-text, and included 24 studies: 16 quantitative and 8 qualitative. Figure 1 details the study selection process. See online supplementary Table S3 for information on excluded studies.

**Fig. 1.**
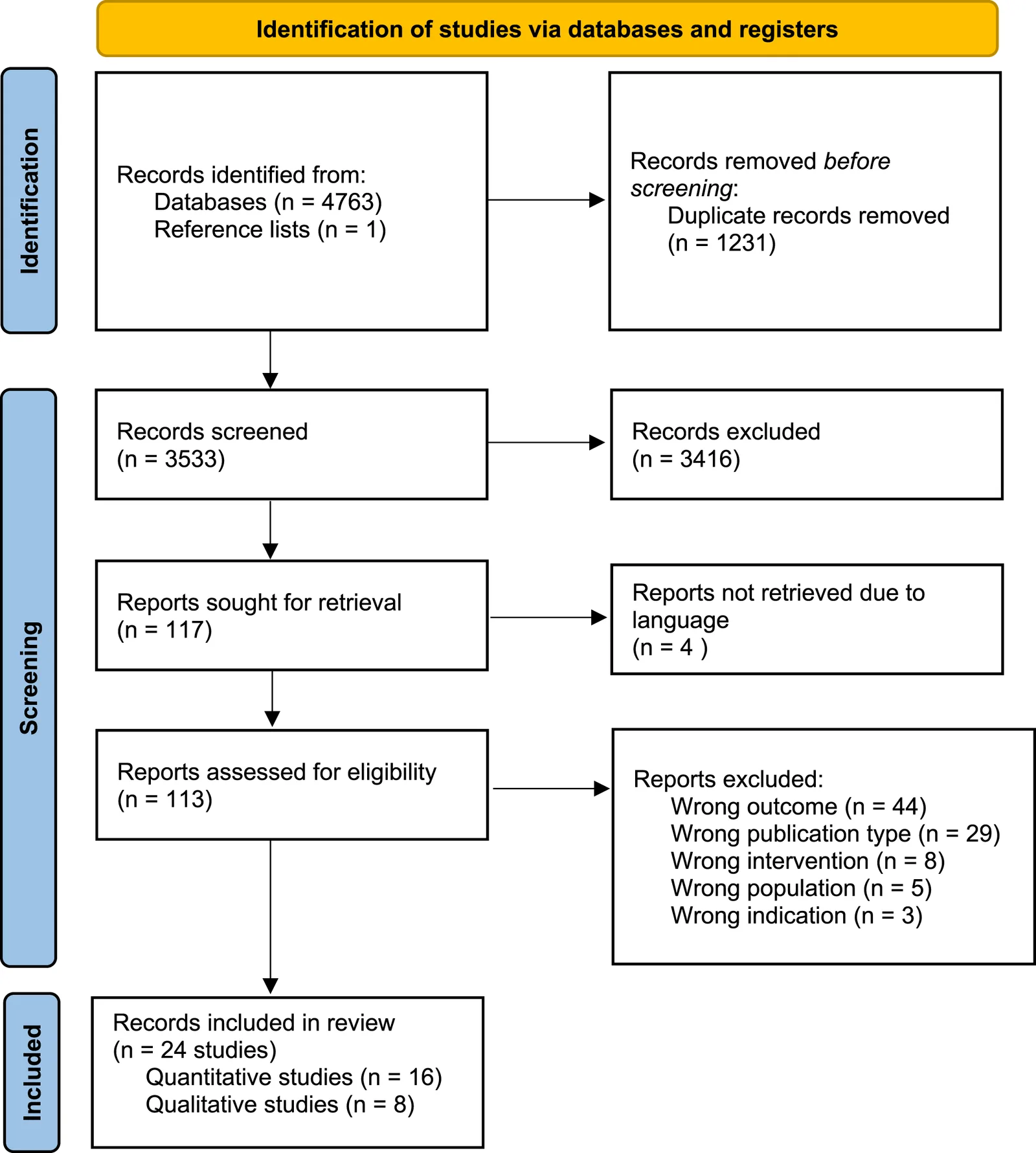
PRISMA flow diagram.

### Characteristics of Included Studies

A summary of the included study characteristics is presented in Table 1, and the qualitative studies are presented in Table 2. A total of 4,394 and 219 participants were included in the quantitative and qualitative studies, respectively. Four studies were conducted on parents [10–13], two on adults born prematurely [14, 15], two on nurses [16, 17], five on physicians [18–22], and three on mixed healthcare professionals [23–26]. Eight studies were conducted among several interest-holder groups [27–33]. Five quantitative studies neglected to report the sex of the participants [10, 19, 20, 24, 29]. Approximately 74% of the participants in the quantitative studies were female, and 55%–100% in the qualitative studies.

**Table 1.** Characteristics of quantitative studies (*n* = 16)

First author, year of publication	Country and setting	Study period	Participants	Gender And Ethnicity	Method of data collection	Outcomes
AlFaleh et al. [27] (2015)	Saudi-Arabia	October 2011 to March 2012	299 HPW (GA 23–28 weeks)	Female: 100%	Interviews	PDA, PDA ligation, severe IVH, and bronchopulmonary dysplasia
	Tertiary, perinatal centers		HRPW (GA 23–28 weeks) and others of ELBW infants	Ethnicity: NR	Computer-based structured decision aid instrument based on a 5-point Likert scale	
Arzuaga et al. [18] (2016)	USA	NR	255 American Muslim physicians	Female: 30%	Questionnaire	Mortality vs. QoL
	Setting NR			Ethnicity: 70% South Asian; 22% Arab or Middle Eastern; 4% White/Caucasian; 1% Black/African American	Non-validated	
Cho et al. [10] (2012)	South Korea	January 2005 to December 2009	390 parents of premature infants (birth weight, <2,500 g; GA, <37 weeks) who went to a neonatal follow-up clinic after NICU discharge at the corrected ages of 0, 4, 8, 12, 18, and 24 months	Gender: NR	Questionnaire	Developmental delay, feeding and nutrition, growth, ophthalmologic problems, skin problems, respiratory morbidity, and behavioral problems at 4, 8, 12, and 18 months
	NICU, hospital			Ethnicity: NR	Non-validated	
de Garis et al. [19] (1987)	Australia	NR	51 neonatal pediatricians	Gender: NR	Questionnaire	QOL, disability, sepsis
	NICU, hospital			Ethnicity: NR	Open-ended questions	
Gallager et al. [16] (2012)	United Kingdom	NR 2007 and NR 2008	36 neonatal nurses from six neonatal units	Gender: 94% female	Q methodology	Mortality vs. general disability or QOL
	NICU, hospital			Ethnicity: NR		
Gallagher et al. (2016) [20]	UK	May and November 2012	25 experienced neonatal doctors (Consultants and Specialty Registrar ≥4 years)	Gender: NR	Q methodology	Severe disability (short- and long term), death, QOL
	Tertiary neonatal care, hospital			Ethnicity: NR	Ranking a set of statements (+6 to −6)	
Gallagher et al. [24] (2022)	UK	February 2020, and April 2021	155 healthcare professionals: neonatal nurses, midwives, neonatologists, and obstetricians	Gender: NR	Q methodology	Mortality, disability
	Perinatal centers, tertiary neonatal care hospital and maternity center			Ethnicity: NR	Ranking a set of statements (+6 to −6)	
Geurtzen et al. [23] (2023)	Netherlands	4 November 2020 to 10 January 2021	297 PHP including obstetricians, neonatologists, obstetric and neonatal nurse, clinical midwives and physician assistants and nurse practitioners	Gender: 82% female	Non-validated questionnaire	Severe behavioral, learning or school problems, the inability to interact and make contact, not being able to live independently, being in pain, not being happy or having QOL, psychiatric problems and long-term lung and gastrointestinal problems. Solitary blindness or deafness
	Perinatal care centers and affiliated referral centres			Ethnicity: NR		
Jaworski et al. [11] (2022)	Canada	July 1, 2018, to July 31, 2019	248 parents of 213 preterm children (GA <29 weeks)	Gender: 70% female	Non-validated questionnaire using rating scales	Development, respiratory health and fragility, nutrition/feeding and growth, language communication, behavior and emotional health, motor development and movements, cognitive and learning skills
	University hospital neonatal follow-up clinics			Ethnicity: Caucasian: mothers 63%, fathers 82%, Black: mother 25%, father 10%, other: mother 12%; father 8%		
LoRe et al. [22] (2023)	USA	December 2019 to February 2020	267 physicians in Neonatology and MFM and their trainees (residents and fellows)	Gender: 74% female	Respondents’ QoL views were assessed in three ways and reported in 4-point forced-choice	QOL
	Neonatology- and maternal and fetal AMC			Ethnicity: NR		
Mitra et al. [29] (2023)	Canada and the United Kingdom	March 3, 2021, to February 10, 2022	9 adults born very preterm (GA <32 weeks) and 31 parents of very preterm infants	Gender: NR	Virtual video-conferenced interviews using structured surveys and semi-structured interview scripts, utilizing a numeric rating scale for assessment	Mortality, severe IVH, chronic lung disease, necrotizing enterocolitis, PDA
	IWK Health NICU, follow-up clinics, and Canadian Premature Babies Foundation organization			Ethnicity: predominantly White (77.5%), with smaller representations of Black (2.5%), Indigenous (5.0%), and other races or ethnicities (15.0%)		
Saigal et al. [13] (2003)	Canada	March 1, 1998, to February 28, 1999	80 pregnant women and 75 mothers of infants who weighed less than 1500 g at birth	Gender: 100% female	Interviews	Disability, hearing, seeing, talking, ADL, pain, cognitive impairments, physical impairments, mental disability
				Ethnicity: White		
	Tertiary NICU and a tertiary care regional perinatal center clinic			Pregnant women: 82%	Standard Gamble technique	
				Mothers of VLBW: 93%		
Schneider et al. [21] (2019)	Germany, Switzerland, and Austria	November 2016 to December 2016	104 HPs of tertiary NICUs	Gender: 23% female	Modified EURONIC questionnaire, unclear if validated	Survival, poor neurological outcome, severe physical disability, severe mental disability
	NICU			Ethnicity: NR		
Streiner et al. [31] (2001)	Canada	NR	146 parents of LBW children, 123 parents of non-LBW children, 98 neonatologists, and 99 neonatal nurses	Gender: NR	Questionnaire with a 6-item self-report developed by Lee et	Survival, severe disability
	Setting NR			Ethnicity: NR		
Synnes et al. [32] (2023)	Canada	April 2021 to June 2021	471 parents of children born preterm, 21 individuals born preterm, 228 HCPs, 14 researchers, 5 trainees, and 14 educators	Gender: Physicians 60% female; Nurses 100% female. Parents predominantly female (86–93%)	Ranking of scenarios and ratings of health states (10-0)	Cognitive impairment, motor impairment, hearing impairment, language delay, motor and cognitive impairment, motor impairment and language delay, cerebral palsy, language delay and cognitive impairment, visual impairment, cerebral palsy and language delay
	Private Facebook groups and online parent-support sites			Ethnicity: NR		
Saigal et al. [30] (1999)	Canada	NR 1993- NR 1995	100 neonatologists, 103 neonatal nurses, 264 adolescents born preterm, 275 parents	Gender: physicians – 40%, nurses – 100%, parents, 89%	Interviews	Cognitive impairment. Vision impairment. Hearing impairment. Language impairments. Mobility impairment. Emotional impairment. Pain
	Tertiary care neonatal referral canters for HCPs, and adolescents			Ethnicity: physician 39% born in Canada; nurses 74% born in Canada; parents 71% born in Canada	Standard Gamble technique of 5 health states	

ADL, activities of daily life; AMC, academic medical center; ELBW, extremely low birth weight EP, extremely premature, GA, gestational age; HP, head physician; HPC, healthcare professionals; HPW, healthy pregnant women; HRPW, high-risk pregnant women; IVH, intraventricular hemorrhage; LBW, low birth weight; MFM, maternal-fetal medicine; NICU, neonatal intensive care unit; NR, not reported; PDA, patent ductus arteriosus; PHP, perinatal healthcare professionals; QoL, quality of life; UK, United Kingdom; VBLW, very low birth weight.

**Table 2.** Characteristics of qualitative studies (*n* = 8)

First author, year of publication	Country and setting	Study period	Participants	Gender and ethnicity	Method of data collection	Methodology
De Boer et al. [34] (2023)	Netherlands	January and February 2022	23 Adults born EP	Gender: 78% female	Focus group	Thematic content analysis
	Setting NR			Ethnicity: NR		
de Boer et al. [12] (2024)	Netherlands	September 2022 and April 2023	19 (26 total parents interviewed) parents of EP children (<23 to <28 weeks)	Gender: 76% female	Interviews (online or face-to-face)	Thematic content analysis
	Online, home, or hospital			Ethnicity: NR		
Einaudi et al. [25] (2012)	France	NR	14 experts who manage EP children (obstetricians, intensive care physicians, neonatal physicians, psychologists and pediatric neurologists	Gender: 71% female	Focus group	Content analysis
	Hospital			Ethnicity: NR		
Lemmon et al. [28] (2019)	USA	NR	69 parents of EP babies and clinicians (attending physicians, fellows, social workers, nurse practitioners, registered nurses)	Gender: 65% parents female, 75% clinician's female	Interviews	Directed content analysis
	Hospital (NICU)			Ethnicity: Parents: African American: 56%, White: 31%, American Indian: 13%. Clinicians: White: 87%, African American: 2%, Hispanic: 11%		
Martone et al. [26] (2025)	Spain	March until November 2023	55 neonatologists, neonatal nurses, gynecologists/obstetricians, and psychosocial workers from different healthcare institutions in Barcelona, and professionals working on the artificial placenta trial	Gender: NR	Interviews, observations (non- participant using “shadowing” technique)	Ethnography, inductive thematic analysis plus social theories
	High-complexity quaternary care hospital and other institutions			Ethnicity: NR		
van Zuuren et al. [33] (2006)	Netherlands	NR	13 healthcare providers: neonatologists, nurses, pastoral workers, social workers	Gender: 77% female	Interviews and observations – “embedded researcher” in order to get familiar with the setting	Grounded theory
	NICU) at a university hospital in the Netherlands			Ethnicity: NR		
Vederhus et al. [15] (2025)	Spain	November 2022 to May 2023	11 individuals being born extreme preterm (≤28 weeks GA) in 1989–1990	Gender: 55% female	Interviews	Reflexive thematic analysis
	Regional university hospital			Ethnicity: NR		
Webb et al. [17] (2014)	USA	NR	16 female nurses working in the NICU	Gender: 100% female	Interviews	Thematic analysis (but phenomenological method design)
	97-bed NICU located in a 259-bed specialty licensed children’s hospital			Ethnicity: NR		

EP, extremely premature; NICU, neonatal intensive care unit; NR, not reported.

Several studies were conducted exclusively in specific countries, including the USA [17, 18, 22, 28], Canada [11, 13, 30–32], Australia [12, 14, 19, 23, 33], Norway [15], France [25], Spain [26], the UK [16, 20, 24], Saudi Arabia [27], and South Korea [10]. Additionally, two studies were conducted in multiple countries [21, 29].

### Risk of Bias

Detailed judgments of the risk of bias in the quantitative studies are presented in online supplementary Table S4. All quantitative studies were rated as having a very or extremely serious risk of bias due to concerns regarding the selection of participants, measurement instruments, and data analysis.

Regarding the qualitative studies, one study was assessed as having minor methodological concerns [17], three as having moderate [25, 26, 33], and four as having serious methodological concerns [12, 14, 15, 28]. Concerns were related to insufficient information on the research design, lack of interest-holder involvement and researcher reflexivity, inappropriate study design, and superficial analysis and reporting of findings. Detailed assessments of the methodological limitations of all the included qualitative studies are provided in online supplementary Table S5.

### Synthesis of Evidence

Detailed results for individual studies are presented in online supplementary Tables S6 and S7 for quantitative and qualitative studies, respectively. Summary of findings tables, including assessments of the certainty of evidence and more details regarding the synthesis of results, are provided in online supplementary Tables S8 and S9, respectively.

#### Survival

A total of 17 studies informed on the value assigned to death and/or survival in relation to other consequences in infants born preterm or very preterm: ten quantitative studies [13, 16, 18–21, 24, 29–31], and seven qualitative studies [12, 14, 15, 17, 26, 28, 33]. Quantitative evidence suggests that, among individuals born preterm and their parents, survival is overall valued more highly than other potential consequences [29], but with substantial variability among parents if put in relation to very severe disability [13, 30, 31]. In general, there were no substantial differences between parents and individuals born preterm (adolescents or adults) in the importance assigned to survival (online suppl. Table S8). Studies examining physicians’ values showed that survival accompanied by severe disability, neurological, mental, or motor impairments, or by severe brain injury, was overall considered the most undesirable outcome, even compared with death [18–20, 24, 30], but with substantial variability between physicians, which was similar to values from nurses [16]. The value of survival did not appear to differ substantially between countries [21]. The certainty of evidence was very low for this outcome across and between the groups. Evidence suggests differences between parents and healthcare professionals in the importance assigned to survival. Parents overall prioritized survival, regardless of birth weight or clinical condition, whereas healthcare professionals assigned a lower value to survival in infants at high risk of severe disability, considering the potential for a long-term burden on families [31].

Qualitative evidence also revealed variability in how both parents and healthcare professionals perceived survival. Some parents strongly prioritized giving their child a chance to survive and hoped to bring their baby home alive [12, 14, 28] although they acknowledged that survival should not be pursued at all costs if the infant is non-viable or treatment is disproportionate, palliative care should be considered [12, 14]. Parents and nurses emphasized the importance of being informed about what an infant must endure to survive after extreme prematurity [14, 17]. When considering care decisions, clinicians emphasized survival, neurologic outcomes, and potential for disability [26, 28, 33], while acknowledging that life-long disabilities are difficult to predict [26, 33]. Nurses questioned whether continuing aggressive treatment and prolonging life is ethical in infants with poor prognoses, reflecting the tension between short- and long-term outcomes [17, 33]. Adults born prematurely and without severe cognitive impairment value overall their survival and express gratitude for the medical interventions and support that enabled it [15]. The evidence for this outcome was of very low certainty (online suppl. Table S9).

#### Quality of Life

Evidence on the value assigned to QoL among parents, healthcare professionals and people born preterm, comes from five quantitative studies [13, 16, 18, 22, 30] and seven qualitative studies [12, 14, 17, 25, 26, 28, 33]. Evidence from quantitative studies overall suggests that a very low QoL is perceived as the most undesirable outcome, but with definitions varying across studies, particularly in early childhood and due to physical, emotional, or cognitive impairments [16, 22, 30]. Experiencing a substantial negative impact on QoL due to a severely disabled health state was overall considered by both healthcare professionals and parents to be worse than death [30]. This evidence suggests that healthcare professionals tend to rate health states associated with severe disability worse than parents and adolescents born preterm, reflected in lower utility scores [16, 22, 30]. Evidence also suggests that preferences for disability-related outcomes among physicians and mothers are influenced by both the severity of the disability and its negative impact on QoL, with more severe disabilities or greater negative impact on QoL being assigned a lower value [13, 30]. Evidence from one study [18] suggests that the value placed on QoL varies among physicians depending on their beliefs: those who view the value of life as independent of QoL tend to assign a lower value to quality-of-life considerations and are more likely to endorse resuscitation against parents’ wishes. Among physicians, attributes such as pain-free living, emotional stability, absence of motor impairment, and independence in self-care are considered the most important contributors to QoL [22]. The certainty of evidence for this outcome was very low across and between groups.

Evidence from qualitative studies shows that the infant’s future QoL was important to many parents, described in terms of “enjoyment,” “happiness,” or the ability “to be and act like others,” such as playing sports, forming relationships, eating independently, and living autonomously [12]. Adults born extremely premature and health care professionals managing extremely preterm born children reported having difficulties in determining a good QoL and the subjective and changing nature of it [14, 25, 26]. Adults born extremely premature emphasized that QoL should not be based solely on physical abilities [14]. In most studies, healthcare professionals expressed deep concerns about the present and future QoL of extremely premature infants. In one study, obstetricians and intensive care physicians prioritized immediate survival over future QoL, considering it unsuitable for decision-making in urgent situations. Some viewed QoL as “dangerousˮ because of its evolving nature and potential influence on medical decisions; however, some neonatologists, particularly those involved in long-term care and more familiar with QoL methods, valued it for guiding their care [25]. Neonatologists from another study considered QoL to be the key criterion for continuing treatment [33]. Healthcare professionals recognized that QoL depends heavily on psychological development and social integration, noting that these psychosocial consequences are often underestimated and require further research [25]. The certainty of this evidence was low.

#### Developmental Impairment

A total of eight studies informed the values assigned to developmental impairment-related outcomes, such as cognitive impairment and cerebral palsy, five quantitative studies [10, 11, 13, 21, 32] and three qualitative studies [14, 17, 28]. In general, the quantitative evidence is of very low certainty and suggests that developmental delay is considered worse compared to other impairment outcomes, such as vision and hearing impairment [13] or respiratory morbidity [11] and behavioral problems [10]. This pattern seems to be consistent across parents, individuals born preterm, and healthcare professionals. Severe cognitive impairments received the lowest values regardless of the measurement instrument used, and together with developmental delay, may be the least preferred outcomes across all groups studied when compared with other health conditions [13, 30, 32]. Among healthcare professionals, neurological and mental disabilities were considered more undesirable than motor disabilities [21]. The combination of several impairments is perceived as worse compared with other outcomes and motor impairments are considered more concerning than cognitive impairments when presented in combination with other impairments and outcomes [32]. Among parents, the importance placed on outcomes may be influenced by their child’s neurodevelopmental impairment and age, as well as by parental education. Parents of children without developmental impairments placed less importance on developmental outcomes than those of children with impairments, while the importance placed to developmental outcomes increased with the child’s age and parental education [11]. The certainty of evidence on this outcome is very low across and between groups.

The qualitative findings are of very low certainty. Evidence from one qualitative study suggests that parents of children born extremely preterm and clinicians value neurologic prognosis differently when considering care decisions for infants [28]. While parents rarely expressed concerns about future neurologic development or cognitive milestones in the hopes of a “normal life,” clinicians’ discussions emphasize neurologic outcomes and the potential for disability. In another study, parents again expressed strong concerns about the chance and nature of potential disabilities their infant may face, questioning what disabilities their child will have and how far they are willing to go for survival, when considering their infant’s future [12]. Physicians and nurses acknowledged the unpredictability of development, noting cases in which initial expectations were proven incorrect [33]. Adults born prematurely described facing cognitive challenges, particularly regarding learning and concentration, creating feelings of being “left behind” academically [15].

Nurses viewed developmental delays and cerebral palsy as one of the most concerning sequelae for extremely preterm infants, who are at a high risk of developing this condition. They reported experiencing moral distress related to the use of life-sustaining technologies when survivors may develop severe cerebral palsy, which often occurs alongside other impairments, such as blindness and deafness [17].

#### Other Impairments (Visual, Hearing or Language Impairment, Motor Impairment, and Behavioral and Social Impairment)

Seven quantitative studies assessed values and preferences associated with various types of impairment [10, 13, 21–23, 30, 32] and three qualitative studies examined different impairments as part of their thematic content analyses [14, 17, 28]. Very low certainty quantitative evidence suggests that, among parents, healthcare professionals, and adults born preterm, different types of impairments may be valued differently. Across all groups, visual impairments were considered the most concerning, followed by language, hearing, motor, and cognitive impairments [32].

Among healthcare professionals, the least desirable long-term outcomes, aside from severe behavioral, learning, and school problems, were inability to interact socially and inability to live independently. Severe mental disability was considered less undesirable than severe motor disability [23]. Severe mental disability is also considered less desirable than severe motor disabilities [21].

Preferences regarding impairments seem to change over time [10, 21, 23]. Among parents, concerns regarding adverse outcomes generally decrease as infants grow older [10]. In contrast, healthcare professionals generally consider outcomes measured after 24 months to be more relevant, noting that earlier outcomes do not reliably predict long-term prognosis [23]. Physicians’ reported values and preferences regarding motor and neurodevelopmental impairments tend to change over time as clinical care evolves [21]. The certainty of evidence for this outcome was very low.

Limited evidence of very low certainty from qualitative studies suggests that nurses consider blindness and deafness as severe long-term impairments that extremely premature infants might face [17]. Clinicians emphasize language development and talking as key developmental milestones in prognostic discussions [28]. In one study, parents’ concerns about their children’s survival were less imminent when walking or talking was the issue [28], but parents expressed concern about motor impairments, such as wheelchair dependence [12]. Adults born extremely preterm often view motor disabilities as particularly limiting, affecting social development, participation in team sports, and engagement in social activities throughout life, even when other physical abilities, such as walking, are preserved [14]. They further reported significant psychosocial challenges including living in constant “survival mode,” struggling to participate in society fully and experiencing bullying and social isolation [14, 15]. Healthcare professionals recognize that these psychosocial consequences are often underestimated [25].

#### Other Clinical Outcomes

A total of ten studies assessed how different interest-holder groups value medical outcomes and complications: seven quantitative studies [10, 11, 19, 22, 27, 29] and four qualitative studies [14, 17, 28, 33]. Evidence is very uncertain regarding the value placed on clinical outcomes, while results from one study suggest that feeding and nutrition concerns are assigned lower values by parents after developmental delay, followed by growth and ophthalmologic and respiratory problems [10]. In contrast, one study suggested that respiratory health is the most concerning outcome and therefore assigned a lower value, followed by feeding, nutrition, and growth problems [11].

Very low certainty evidence also suggests that parents, pregnant women, and individuals born preterm assign a value to intraventricular hemorrhage, considering it the most severe and undesirable outcome after death, followed by necrotizing enterocolitis, patent ductus arteriosus, and chronic lung disease/bronchopulmonary dysplasia [27, 29]. Evidence from studies assessing physicians’ preferences [19] suggests that severe brain damage has the highest value, followed by congenital deformity, permanent disability, irreversible respiratory failure, and sepsis. Other studies of healthcare professionals [22, 23] provide evidence that pain is considered the most unfavorable symptom, followed by lung and gastrointestinal problems, with very low certainty of evidence.

The evidence was inconsistent within groups, which increased uncertainty. Parents’ preferences may differ across families, and among physicians based on seniority and specialty [11, 23]. For instance, parents of children with more severe developmental impairments may rate nutrition and growth problems as greater concerns, while considering respiratory illness and fragility as lesser concerns, compared with parents of children without severe impairment. Evidence of very low certainty also suggests minimal variability in judgments about the importance of intraventricular hemorrhage, but substantial differences for necrotizing enterocolitis, patent ductus arteriosus, and chronic lung disease among parents and individuals born preterm [29].

Qualitative evidence suggests that, among nurses caring for extremely premature infants, severe sequelae, such as infections, sepsis, intraventricular hemorrhage, respiratory distress syndrome, and blindness, are among the most concerning outcomes. Consequently, these were assigned lower values compared to other clinical outcomes, except for cerebral palsy and developmental delays [17]. To these nurses, pain or suffering caused by life-prolonging treatment devices was also a major concern [17]. Physicians were more concerned about possible future damage that by their treatment may induce. However, they also acknowledged the moral challenge of weighing the benefits against possible harm [28, 33]. Furthermore, parents were concerned about long-term feeding dependencies, including tube feeding and the inability to eat independently, which is a very undesirable outcome affecting QoL [14]. The certainty of evidence for these outcomes was very low.

## Discussion

### Summary of Main Results

The review identified values and preferences for a wide range of long- and short-term outcomes in preterm infants. These outcomes spanned survival, long-term disability, and short-term clinical conditions, and were assessed across parents, healthcare professionals, and individuals born preterm.

Overall, the evidence was of very low certainty, primarily due to risks of bias, including limitations in the validity of measurement instruments, heterogeneity in study designs, and variability in participant selection. This evidence suggests that survival is consistently valued as the most important outcome by parents and individuals born preterm. In contrast, healthcare professionals placed a relatively lower value on survival when associated with a high risk of severe disability, but with significant variation within each group. Severe disability is generally considered less desirable than survival; among disability-related outcomes, mental and neurodevelopmental impairments are consistently rated as the least preferred. In the short term, intraventricular hemorrhage was identified as the most severe and undesirable condition. However, preferences regarding other outcomes, such as respiratory morbidity, feeding and growth problems, and sensory impairment, varied across the studies and groups.

Differences in valuation may explain the variation between interest-holder groups. Parents tended to focus more on survival and, in some cases, expressed limited concern about long-term neurodevelopmental outcomes, whereas healthcare professionals emphasized neurologic prognosis, disability, and QoL. Adults born preterm highlighted the long-term negative impact of motor and physical impairments on social participation and QoL, underscoring perspectives that may be underrecognized by other groups. Qualitative evidence, despite its low certainty, further illustrated that suffering and pain, particularly in the context of intensive care and end-of-life situations, were major concerns among healthcare professionals, especially nurses.

Preferences seem to be influenced by the severity of outcomes and their perceived negative impact on QoL, with more severe impairments consistently assigned lower values. There was variability within interest-holder groups, suggesting that values and preferences are not uniform and may differ between families, individuals, and professionals, as well as across clinical contexts. Additionally, some outcome preferences appeared to change over time, both in the relative importance assigned to outcomes and in the types of impairments considered most concerning at different stages of a child’s development.

Because the evidence is very uncertain, the value judgments identified, particularly regarding the trade-offs between survival and severe disability, should be interpreted with caution. These findings are often based on group averages and are likely to be context-sensitive, highlighting the importance of individualized and context-specific decision-making.

This review provides the most comprehensive synthesis to date of the evidence on values and preferences related to outcomes for preterm infants. While the findings may inform clinical decision-making, guideline development, and the education of health professionals regarding the values of treatment and their potential differences between groups, they should be considered in light of the uncertainty of the evidence and the substantial variability both between and within interest-holder groups.

### Applicability of Our Findings

The evidence covers the major interest groups such as parents, different healthcare personnel, as well as individuals born preterm. The latter was included in only a minority of studies and covered only some of the outcomes. Further evidence from this group would enhance the applicability of the evidence and complement perspectives from parents and healthcare professionals. Only two studies were conducted in settings outside of Europe, North America, and Australia (i.e., Saudi Arabia and South Korea), most were conducted in academic settings, and the values of certain outcomes may differ from our findings due to cultural or other factors. Furthermore, the values and preferences differed for certain outcomes across interest-holders, which is valuable information to have in the clinical encounter and for the development of guidelines.

### Strengths and Limitations

The review expands upon and complements prior findings by primary studies by integrating them with similar work to reach more confident conclusions, and by including and exploring additional populations, settings, and outcomes while also incorporating the certainty of the evidence. The review also includes values elicited using different methods, provide a broader overview of what is currently known and identify important gaps and priorities for future research. We followed the standard Cochrane methodology for the review, including a broad search within several databases; however, we did not search the gray literature or use other supplementary methods to identify further studies. Furthermore, we applied newly developed methods for the synthesis and assessment of studies on values and preferences for outcomes, for bias assessment (RoB-VALU; CAMELOT) and for certainty of evidence (GRADE), increasing the reliability and validity of our findings. A potential limitation of applying these newly created methods is that, so far, there has been no widespread application of these methods, and the availability of detailed guidance based on broad user feedback is not available, which could lead to discrepancies between assessments or reliability or validity concerns if issues are present. We partly mitigated this by obtaining consensus among the assessors for all these assessments.

This review’s priority was to focus on all relevant and reported outcome values for multiple interest-holders, rather than just on one or a small subset of outcomes. Further reviews and further primary studies could have a broader scope or focus more deeply on specific outcomes. Finally, the evidence synthesis depends on the quality and reporting of the available evidence, which requires interpretation by the authors. Because there are substantial uncertainties within the available evidence, the presented synthesis itself includes uncertainty. We aimed to mitigate this by reporting our certainty of the evidence in connection with each result, and the reported results should always be interpreted in light of this.

### Certainty of the Evidence and Research Implications

The overall certainty of the evidence was very low, primarily due to inconsistency across study results and a significant risk of bias in the included studies. Most studies relied on instruments of uncertain validity, used heterogeneous methods, and often reported results descriptively, limiting opportunities for in-depth analysis or synthesis.

To improve the certainty of future evidence, instruments and methods used to measure values regarding neonatal and long-term outcomes should be validated and may include a neonatal core outcomes set, which could reduce variability across studies. Attention should be paid to assessing the relative importance of different outcomes, with clearly defined outcomes, explicit descriptions of consequences, and specified timeframes.

Future research should also focus on larger and more representative groups where evidence related to values and preferences is needed, such as among people born very preterm while recognizing the specific enrollment and ethical considerations related to the group compared to parents and healthcare professionals, to ensure that participants reflect the diversity of the underlying population. Recruitment strategies should be inclusive, to avoid selection bias that may limit the generalizability of findings. Using consistent methods across studies would also facilitate future syntheses, facilitating statistical synthesis across studies, and enabling more robust comparisons of how values and preferences vary between interest-holder groups and across different outcomes over time.

### Agreements and Disagreements with Other Studies or Reviews

A previous scoping review [34] of parental perspectives on decision-making at the limits of viability also highlighted the value of longer term outcomes of disability and impairments compared to survival, identifying variability among parents. Our review focused on outcomes beyond children at the limit of viability and values from other interest-holder groups, expanding the applicability of outcomes to new settings and people.

Furthermore, a broad qualitative evidence synthesis [35] examining the families’ view of the care of preterm infants also identified survival as the main outcome of importance, but at the same time showed that shorter term outcomes were more often mentioned than longer term outcomes, and that both short- and longer term outcomes are considered important in the care of preterm infants.

## Acknowledgments

During the preparation of this manuscript, we used ChatGPT and Copilot to improve readability, grammar, and language. We reviewed and edited the content as needed and take full responsibility for the content in the manuscript.

## Statement of Ethics

Statement of Ethics is not applicable because this study is based exclusively on published literature.

## Conflict of Interest Statement

Martin Ringsten, Kenneth Färnqvist, Vera Wachtmeister, María Ximena Rojas-Reyes, and Isolde Sommer: no conflict of interest to declare. Katarzyna Wróblewska-Seniuk: works as a physician (MD, PhD) at the II Department of Neonatology of Poznan University of Medical Sciences in Poznan, Poland. She declares no conflicts of interest. Matteo Bruschettini: conducts systematic reviews and develops guidelines for interventions used to guide treatment for preterm infants. He declares no conflicts of interest.

## Funding Sources

This work was supported by Forska Utan Djurförsök and an ALF (Avtal om Läkarutbildning och Forskning) grant.

## Author Contributions

Martin Ringsten: conceptualization, investigation, formal analysis, methodology, data curation, writing – original draft, and project administration. Kenneth Färnqvist and Vera Wachtmeister: investigation, formal analysis, and writing – review and editing. Katarzyna Wróblewska-Seniuk and Matteo Bruschettini: conceptualization, methodology, and writing – review and editing. María Ximena Rojas: investigation, conceptualization, methodology, writing – original draft, and writing – review and editing. Isolde Sommer: conceptualization, investigation, methodology, data curation, formal analysis, and writing – original draft.

## Data Availability

All data generated or analyzed during this study are included in this article and its supplementary material files. Further inquiries can be directed to the corresponding author.
